# Resilient Communication for Software Defined Radio: Machine Reasoning and Electromagnetic Spectrum Evaluation

**DOI:** 10.3390/s25061826

**Published:** 2025-03-14

**Authors:** Sergey Edward Lyshevski, Richard Buckley, Christopher Feuerstein

**Affiliations:** 1 Department of Electrical and Microelectronic Engineering, Rochester Institute of Technology, Rochester, NY 14623, USA; 2 L3Harris Technologies, Rochester, NY 14610, USAchris.feuerstein@l3harris.com (C.F.)

**Keywords:** electromagnetic spectrum, interference, jamming, machine reasoning, software defined radio

## Abstract

This paper investigates evaluation methodologies and machine reasoning schemes to analyze dynamic electromagnetic spectrum. We research practical and scalable classification of radio frequency signals across high frequency, very high frequency, ultra high frequency and super high frequency bands. The multi-band software defined radio, software defined mobile networks, and global navigation system receivers accomplish reconfigurable communication. Resilient communication, as well as high-fidelity analysis of extreme and congested electromagnetic spectra, are open problems due to challenges in classification of interference, distortions and adaptive jamming from spatially distributed transmitters and jammers. This paper documents high-fidelity characterization and dynamically reconfigured machine reasoning schemes to ensure the cognitive capabilities of communication systems. Low-fidelity experimental studies substantiate the spectrum evaluation methodology and demonstrate results.

## 1. Introduction

Compact, modular and programmable multi-band multi-channel radios with tunable filter accessories have been commercialized. In congested, contested and constrained electromagnetic spectrum (EMS), advanced radios should ensure resilient communications across high frequency (HF), very high frequency (VHF), ultra high frequency (UHF) and super high frequency (SHF) bands, e.g., in the 1 MHz to 30 GHz frequency range [1,2]. The software defined radio (SDR) paradigm has emerged and is effectively used across all domains. Studies are conducted in land [3,4], air [5,6], sea [7,8], space [6,7] and cyber [9,10] domains. The SDR and communication platforms are commercialized with field-programmable gate arrays (FPGA) and system on a chip (SoC). Advanced algorithms, edge computing and parallelism empower adaptive signal processing in near real time [11,12]. There are a number of challenges and open problems in the design of SDRs [13,14,15,16]. The 6G cellular technology requires new solutions, which amplify the use of multi-band SDRs [17,18]. An all-domain command and control framework and 6G wireless technology imply the design of next-generation communication platforms to ensure communication superiority and seamless information sharing [19]. Robust multi-band wireless cellular technologies with learning capabilities [20,21] are emerging. A consistent integration of a frontend module with a variable-gain amplifier and FPGA enables high-performance processing, networking, robustness, speed, control, filtering, reconfigurability, signal conversion and interfacing.

Frequency hopping spread spectrum (FHSS), direct sequence spread spectrum (DSSS) and chirp spread spectrum (CSS) schemes have been deployed. The EMS must be evaluated and characterized in congested and adversarial environments [19,20,21]. A number of publications focus on classification of modulation, interference and jamming. The analog and digitally modulated RF signals were classified in [22,23,24,25] by using recurrent and convolutional neural networks. Publications [22,23,24,25] and the references therein report notable inconsistencies and misclassifications, even for low signal-to-noise ratio (SNR) RF signals for different modulations. The achievable accuracy in low-fidelity studies reaches 90%. The misclassification was due to the use of single-domain attributes, dataset inadequacy, and models training on truncated and inconsistent attributes and features. Measurable descriptive spectral and probability time-varying characteristics should be devised and applied. Otherwise, the RF signals are found to be indistinguishable and imperceptible. Paper [26] investigates orthogonal frequency division multiplexing multi-carrier modulation. Using SDR, the authors study spectrograms for signals in subbands, characterized by frequency, strength, threshold and SNR. The machine learning classifier yields inadequate accuracy in detection and classification of the carrier signals, as well as inconsistent detections of the spot, sweep and barrage jamming. An alternative concept is studied in [27]. The in-phase and quadrature IQ imbalance transmitter *signature* was proposed as a feature. Using neural networks, focused studies were carried out, reporting challenges and impediments [27].

The theoretical studies [28,29] should be substantiated and verified. We conduct fundamental research with experimental validation. The reported proof-of-concept cognitive SDR- and RFSoC-centric spectrum characterization schemes and algorithms offer novel solutions. Machine reasoning aggregates learning and evaluation by means of descriptive, prescriptive and predictive analyses. These enable pattern recognition, detection and clustering. The reasoning algorithms are developed by applying the *Büchi automata* scheme, temporal logics and neural networks using finite-dimensional vector spaces of attributes and features.

## 2. Radio Frequency Signals in Temporal, Spectral and Probability Domains

RF signals exhibit measurable descriptive attributes from which one may derive predictive and prescriptive features in temporal, spectral and probability domains. Evaluation, characterization, reasoning, pattern recognition and other tasks can be accomplished by means of data mining and machine learning.

Data-driven independent, correlated and aggregated features should explicitly describe the information content in multiple domains, supporting the multiple-level feature and metrics extraction. The domain-specific attribute and feature selection, mining, factorization, ranking and dimensionality reduction aim to support robust high-fidelity evaluation and benchmarking using performance metrics.

We consider signal processing, data management and information flow as $(\underset{\begin{array}{l} \;\mathrm{RF}\\ \mathrm{Signal} \end{array}}{x(t)},\underset{\begin{array}{l} \mathrm{Measured}\\ \mathrm{RF}\;\mathrm{Signal} \end{array}}{\bar{x}(t)},\underset{\begin{array}{l} \mathrm{Digitized}\\ \mathrm{RF}\;\mathrm{Signal} \end{array}}{\bar{x}[n]},\underset{\begin{array}{l} \mathrm{Filtered}\\ \mathrm{Output} \end{array}}{x[n]})\overset{\mathrm{Attributes}}{\underset{\begin{array}{l} \;\mathrm{Features}\;\mathrm{in}\;\\ \;\mathrm{Time},\;\mathrm{Spectral}\;\mathrm{and}\\ \mathrm{Probability}\;\mathrm{Domains} \end{array}}{\Rightarrow }}\left[\begin{array}{l} \;\mathrm{Spectrum}\\ \;\mathrm{Evaluation}\;\mathrm{and}\\ \mathrm{Characterization} \end{array}\right]$. The RF signal *x*(*t*) is a superposition of the carrier (source) signal, interference, jamming, multisource distortions, etc. The signal *x*(*t*) is filtered by the front-end filter [30,31,32,33], digitized by the analog-to-digital converter (ADC), and filtered by the high-order adaptive bandpass infinite impulse response (IIR) or finite impulse response (FIR) filters [34,35,36]. Signals $\left(x(t),\bar{x}(t),\bar{x}[n],x[n]\right)$ are perturbed and superimposed with multispectral multisource noise n, as well as different origin errors ε and distortions ∆. Signal processing implies amplification, buffering, isolation, pre-filtering, analog-to-analog and analog-to-digital conversions, range matching, interfacing, filtering, estimations, etc.

Measured and processed $\left(\bar{x}(t),\bar{x}[n],x[n]\right)$ are perturbed by noise, distortions and errors (n, ∆, ε) due to antenna arrays, sensors and components heterogeneity and nonlinearities, temperature and environmental sensitivity, sampling and quantization errors, etc. The observed stochastic and deterministic impairments yield measurement errors, reduced throughput, degraded data quality, and information losses. Nonlinear dependences and statistical models of pertained random and deterministic processes are complex. The superposition principle is not applicable.

### 2.1. Adaptive Bandpass Filters Design

There are multiple frequency bands and subbands. The front-end filters are designed as reported in [30,31,32,33]. In SDRs, the RF signal is amplified by an adaptive variable-gain low-noise amplifier, and filtered by a programmable analog front-end filter. The digitized signal is filtered by the Parks–McClellan, Chebyshev and other bandpass filters [34,35,36]. Adaptive high-order FIR filters are implemented by the reconfigurable FPGA and RFSoC. For given bands with Ω∈[*ω*_min_, *ω*_max_], one specifies the following: (i) The cut-off, passband and stopband edge frequencies (*ω_c_*, *ω_p_*, *ω_s_*); (ii) The passband, stopband and sampling frequencies (*f_p_*, *f_s_*, *f_T_*); (iii) The passband and stopband ripples (*δ_p_*, *δ_s_*). Within the RF bands and signal modalities, digital filters are designed considering the multispectral noise and distortions.

We find the filter structure as linear, nonlinear or as rational difference and recurrence equations with constant or varying coefficients. Define homogeneity and heterogeneity measures $\left(M_{H},M_{L}\right)$ within the specified (*ω_c_*, *ω_p_*, *ω_s_*, *δ_p_*, *δ_s_*). The adaptive filters with varying structure *S*(*M*, *N*) and coefficients *C*(*a_l_*, *b_k_*) are computed and reconfigured in real-time using $\left(M_{H},M_{L}\right)$. The filter design yields the order and degree (*M*, *N*). The constraints *g*(·) are the filter complexity and *S*(*M*, *N*)_max_, which indicate robustness, sensitivity and latency. An adaptive filter with the output *y*[*n*] is designed by solving the constrained minimax problem [37,38]:

(1) $$\underset{M_{H}}{\max}\underset{M_{L}}{\min}\left[M_{H}(x,S(\cdot ),C(\cdot )),M_{L}(x,S(\cdot ),C(\cdot )),g(\cdot )\right],(t,\Omega ).$$

Using a finite set of *design variables*, including *S*(*M*, *N*) and *C*(*a_l_*, *b_k_*), quantitative objective measures $\left(M_{H},M_{L}\right)$ as well as constraints, an adaptive filter with varying structure and coefficients (*a_l_*, *b_k_*) is designed as:

(2) $$y[n]=\sum_{k=0}^{N}b_{k}x[n-k]-\sum_{l=1}^{M}a_{l}y[n-l]=b_{0}x[n]+b_{1}x[n-1]+\ldots +b_{N}x[n-N]-a_{1}y[n-1]-\ldots -a_{M}y[n-M],\;G_{S(M,N),C(a_{k},b_{k})}(z)=\frac{Y(z)}{X(z)}=\frac{\sum_{k=0}^{N}b_{k}z^{-k}}{1+\sum_{l=0}^{M}a_{l}z^{-l}},\sum_{n=0}^{\infty }\left|g[n]\right|<\infty ,*z_{i}*\;<\;1.$$

### 2.2. Periodogram and Spectrogram

To analyze the power and frequency content of *x*(*t*), compute the descriptive spectral metrics of a finite-duration discrete-time *x*[*n*] with *N* samples. The discrete Fourier transform (DFT) is expressed as:

(3) $$X[k]=\sum_{n=0}^{N-1}x[n]e^{-j\frac{2\pi }{N}kn},\;k\;=\;0,\;1,\;2,\;\ldots ,\;N-1,\;x[n]=\frac{1}{N}\sum_{k=0}^{N-1}X[k]e^{j\frac{2\pi }{N}kn},\;n\;=\;0,\;1,\;2,\;\ldots ,\;N-1.$$

The periodogram yields the power spectral density (PSD) of sequences *x*[*n*] as the squared magnitude, given as:

(4) $$S_{xx}[k]={\left.S_{x}(\omega )\right|}_{\omega =\frac{2\pi k}{N}}=\frac{1}{N}{\left.{\left|X(e^{j\omega })\right|}^{2}\right|}_{\omega =\frac{2\pi k}{N}}=\frac{1}{N}{\left|X[k]\right|}^{2}=\frac{1}{N}{\left|\sum_{n=0}^{N-1}x[n]e^{-j\frac{2\pi }{N}kn}\right|}^{2},\;k\;=\;0,\;1,\;2,\;\ldots ,\;N-1.$$

Examine temporal and spectral modalities. We split *x* into overlapping segments and use the window *w*[*k*]. For *x*[*n*] of length *N*, consider consecutive segments of length *m*, *m*<<*n*. We define a mapping in |^m×(*N*−m+1)^, with the rows and columns indexed by time as [*x*[0], *x*[1], …, *x*[*m*−1]]*^T^* and [*x*[1], *x*[2], …, *x*[*m*]]*^T^*, e.g., the first and second columns. The spectrogram of *x*[*n*] comprises the DFT columns with the rows indexed by frequency, while the columns are indexed by time. The spectrograms for *x*[*n*] provide a time-frequency representation of the RF signals. That is, *S_xx_*(*ω*) yields *P_xx_*(*t*,*ω*). The resulting *P_xx_*(*t*,*ω*) is used as the feature to characterize a spectrum and signals.

The spectral moments are computed using the PSD, given as:

(5) $$\mu_{t,\omega }^{\left(r\right)}=\frac{1}{\int {\left|X(t,\omega )\right|}^{2}dt}\int \omega^{\left(r\right)}{\left|X(t,\omega )\right|}^{2}d\omega ,\;{\left|X[k]\right|}^{2}={\left|\sum_{n=0}^{N-1}x[n]e^{-j\frac{2\pi }{N}kn}\right|}^{2},$$

where $\mu_{t,\omega }^{\left(r\right)}$ are the conditional temporal-spectral moments.

The experimental studies are reported in Section 4 for signals in the subbands in the congested spectrum. The periodogram, spectrogram and spectral moments are computed and documented.

### 2.3. Statistical Model and Descriptive Statistics

For a random process, a pair of observations *S* and distributions *D* yields a statistical model *R_X_* as:

*R_X_* = (*S*, *D*). (6)

A probability distribution *D*(·) is found and parameterized as:

*D* = {*p_X_*(*x*:*ϕ*), *F_X_*(*x*:*ϕ*), *ϕ*0Φ}, (7)

where *p_X_*(*x*) is the probability mass function (pmf), $\sum_{x\in X}p_{X}(x)=1$, *p_X_*(*x*) ≥ 0; *F_X_*(*x*) is the cumulative distribution function (cdf); Φ is the set of parameters, Φ0|.

The RF signals are not stationary. The distributions and parameters in (7) vary. Analyze the centered stochastic processes (*x*(*t*), *x*[*n*]) which exhibit patterns and dependences. These (*x*(*t*), *x*[*n*]) are not statistically random. The informative features and observed indicators are detected, extracted and computed, yielding dependences, causality and correlations. To find the distribution, compute the histogram, descriptive pair (*F_X_*(*x*), *p_X_*(*x*)) and probabilities **P**(·), given as:

(8) $$F_{X}(x)=P(X\leq x)=\sum_{x_{i}\leq x}P(X=x_{i})=\sum_{x_{i}\leq x}p_{X}(x_{i}),p_{X}\left(x\right)=P\left(X=x\right),p_{X}\left(x_{k}\right)=P\left(X=x_{k}\right),k=1,2,\ldots .$$

The expected value **E**[*X*], or mean *μ*, is calculated as:

(9) $$\mu =E[X]=\sum_{i}x_{i}p_{X}(x_{i}).$$

The standard moment of degree *r* is calculated as:

(10) $$\begin{array}{c} {\bar{\mu }}_{r}=\frac{\mu_{r}}{\sigma^{r}}=\frac{E[{\left(X-\mu \right)}^{r}]}{{\left(\sqrt{E[{\left(X-\mu \right)}^{2}]}\;\right)}^{\begin{array}{l} r \end{array}}}\\ {\bar{\mu }}_{1}=\frac{\mu_{1}}{\sigma^{1}}=\frac{\mu -\mu }{\sqrt{E[{\left(X-\mu \right)}^{2}]}}=0,\;{\bar{\mu }}_{2}=\frac{\mu_{2}}{\sigma^{2}}=\frac{E[{\left(X-\mu \right)}^{2}]}{E[{\left(X-\mu \right)}^{2}]}=1,\\ {\bar{\mu }}_{3}=\frac{\mu_{3}}{\sigma^{3}}=\frac{E[{\left(X-\mu \right)}^{3}]}{(E{\left[{\left(X-\mu \right)}^{2}\right)}^{3/2}},\;{\bar{\mu }}_{4}=\frac{\mu_{4}}{\sigma^{4}}=\frac{E[{\left(X-\mu \right)}^{4}]}{(E{\left[{\left(X-\mu \right)}^{2}\right)}^{2}}, \end{array}$$

where ${\bar{\mu }}_{3}$ and ${\bar{\mu }}_{4}$ are the distribution skewness (asymmetry) and kurtosis.

To characterize the instantaneous variations, the conditional temporal-spectral moments $\mu_{t,\omega }^{\left(r\right)}$ (5) and standard moments ${\bar{\mu }}_{r}$ (10) are used. Spectral density estimation, nonparametric statistics, as well as other probabilistic and correlation analyses yield statistical and deterministic dependences.

*Stationarity of RF Processes*—A continuous-time random process {*x*(*t*),*t*∈|} is stationary if for (*t*_1_, *t*_2_, …, *t_N_*)∈|, the joint probability distribution of {*x*(*t_i_*)} and {*x*(*t_j_*)} is the same as the joint distribution of {*x*(*t_i_*+*τ*)} and {*x*(*t_j_*+*τ*)}, *τ*∈|. For the covariance stationary processes, the mean and autocorrelation function are the same, and the cross-covariance function depends only on the time difference (*t*_1_ − *t*_2_). That is, **E***_X_*(*t*) = **E***_X_*(*t*+*τ*). The RF signals are not stationary.

Table 1 reports the attributes and features.

## 3. Interpretable Machine Reasoning

Consider a conditional probability model. A scalable naïve Bayes linear probabilistic classifier assumes that the features are conditionally independent for given classes *C_k_*. The number of parameters is linear in the number of variables *x_i_*, yielding indicators and predictors. The training algorithm implies a maximum likelihood estimation of parameters for an assumed probability distribution, given the observed data.

The conditional probability is found using the joint probability $p(C_{k},x_{1},\ldots ,x_{n})$ as:

(11) $$p(C_{k}\left|x\right.)=\frac{p(C_{k})p(x\left|C_{k}\right.)}{p(x)},\;p(C_{k},x_{1},\ldots ,x_{n})=p(x_{1}\left|x_{2},\ldots ,x_{n}\right.,C_{k})\times \ldots \times p(x_{n-1}\left|x_{n}\right.,C_{k})p(x_{n}\left|C_{k}\right.)p(C_{k}).$$

Assume that *x* = [*x*_1_, …, *x_n_*] are mutually independent, and conditional to the class *C_k_*. That is, $p(x_{i}\left|x_{i+1},\ldots ,x_{n}\right.,C_{k})=p(x_{i}\left|C_{k}\right.)$. From (11), the conditional distribution over *C_k_* is:

(12) $$p(C_{k}{\left|x\right.}_{1},\ldots ,x_{n})=\frac{p(C_{k})}{\sum_{k}p(C_{k})p(x_{1}\left|C_{k}\right.)}\prod_{i=1}^{n}p(x_{i}\left|C_{k}\right.),\prod_{i=1}^{n}p(x_{i}\left|C_{k}\right.)=p(x_{1}\left|C_{k}\right.)\times \ldots \times p(x_{n}\left|C_{k}\right.).$$

Machine reasoning uses an independent probability model. The maximum a posteriori implies minimization of misclassification probability. Using the likelihood $p(x\left|C_{k}\right.)=\prod_{i=1}^{n}p(x_{i}\left|C_{k}\right.)$, the Bayes classifier implies assigning labels $\hat{l}=C_{k}$, given as:

(13) $$\hat{l}=\underset{k\in \{1,\ldots ,K\}}{\arg\max}p(C_{k})\prod_{i=1}^{n}p(x_{i}\left|C_{k}\right.).$$

Apply the Jaccard index to evaluate the statistical similarity and matching. The Bayes theorem is $p(A\left|B\right.)=\frac{p(B\;\left|A\right.)p(A)}{p(B)},\;p(B)\neq 0$. For a conditional probability, $p(A\left|B\right.)=\frac{p(A\cap B)}{p(B)}$. Consider a finite set of mutually exclusive and jointly exhaustive events. The law of total probability for conditional probabilities is:

(14) $$p(A\left|C\right.)=\frac{p(A,C)}{p(C)}=\frac{\Sigma_{n}p(A,B_{n},C)}{p(C)}=\underset{n}{\Sigma }p(A|B_{n},C)p(B_{n}|C),\;p(C)\neq 0.$$

Assuming that *C* is independent of any {*B_n_*:*n* = 1,2,…}, one has the following:

(15) $$p(A\left|C\right.)=\underset{n}{\Sigma }p(A|C,B_{n})p(B_{n}),p(\mathrm{Class}\left|\mathrm{Features}\right.)=\frac{p(\mathrm{Features}\left|\mathrm{Class}\right.)p(\mathrm{Class})}{p(\mathrm{Features})}.$$

One obtains:

(16) $$p({\mathrm{feature}}_{1},\ldots ,{\mathrm{feature}}_{n}\left|\mathrm{Class}\right.)=\prod_{i=1}^{n}p({\mathrm{feature}}_{i}\left|\mathrm{Class}\right.).$$

The Jaccard index is the measure of similarity between finite sets and is defined as the intersection divided by the union of the sample sets. Consider a finite union of sets (*A*_1_, …, *A_n_*), *A*_1_∪ … ∪*A_n_*. Using $\cup_{i=1}^{n}A_{i}$ and intersections $\cap_{i=1}^{n}A_{i}$, we have:

(17) $$J(A_{i})=\frac{\left|\cap_{i=1}^{n}A_{i}\right|}{\left|\cup_{i=1}^{n}A_{i}\right|},\;0\leq J(A_{i})\leq 1.$$

For (*A*, *B*), one obtains:

(18) $$J(A,B)=\frac{\left|A\;\cap B\right|}{\left|A\;\cup B\right|}=\frac{\left|A\;\cap B\right|}{\left|A\right|+\left|B\right|-\left|A\;\cap B\right|},\;0\leq J(A,B)\leq 1.$$

If there is no intersection, then *J*(*A*, *B*) = 0. The Jaccard distance is:

(19) $$d_{J}(A,B)=1-J(A,B)=\frac{\left|A\;\cup B\right|-\left|A\;\cap B\right|}{\left|A\;\cup B\right|}.$$

For the binary features, define the following: (1) (*M*_11_, *M*_00_) are the total number of features when (*A*, *B*) = 1 and (*A*, *B*) = 0; (2) *M*_01_ is the total number of features when the number of attributes of *A* and *B* are 0 and 1; (3) *M*_10_ is the total number of features when the number of attributes of *A* and *B* are 1 and 0, respectively. One then obtains:

(20) $$J=\frac{M_{11}}{M_{11}+M_{10}+M_{01}},\;d_{J}=1-J=\frac{M_{10}+M_{01}}{M_{11}+M_{10}+M_{01}},\;0\leq J\leq 1,\;M_{11}+M_{00}+M_{01}+M_{10}=n.$$

Using the *Büchi automata*, which admits the *model checking*, the interpretable machine reasoning scheme is given as follows [37,39]:

(21) *B* = (*S*, *s*(*s*_0_,*s_f_*), *A_b_*, τ, *F_s_*),

where *S* is a finite set of identifiers and indicator states *s*(*t*) with initial and final states (*s*_0_,*s_f_*); *A_b_* is the fine alphabet set of propositional logic; τ is the transition function; *F_s_* is a finite set of the admissible conditions.

The *Büchi automata* supports reasoning in temporal, spectral and probabilistic domains. The temporal logic neural network is designed using the descriptive dynamic logic [37] with predicates *p_p_***∈***P*, variables, non-logical objects, finite-state verification and deductive system with logic operators, such as conjunction, disjunction, negation, conditions and logical connectivity in multiple domains.

## 4. Experimental Studies: Electromagnetic Spectrum and RF Signals Evaluation

The spectrum bands in the MHz to GHz frequency range were investigated. The experimental studies were conducted using the ADALM-PLUTO (Analog Devices Inc.), HackRF One (Great Scott Gadgets), RTL-SDR (Nooelec Inc.) and SDRPlay (SDRPlay Ltd.) SDRs. We evaluated the VHF and UHF bands. In particular, study the LTE-450 considering 450–454 MHz band, as well as the Long Range Wide Area Network (LoRaWAN) EU868 863–870 MHz, and, US915 902–928 MHz bands.

Different front-end filters have been investigated [30,31,32,33]. The receiving antenna is connected to the RLC-filters [30,31], or surface and bulk acoustic wave filters [32,33]. The experimental results in [30,31,32,33] demonstrate the exceptional performance for the VHF and UHF subbands. Investigated SDRs with tunable multi-band variable-gain bandpass passive filters with high-Q RLC components to ensure optimal reception. These SDRs admit data fusion of the measured raw modulated and demodulated IQ signals. The synchronous serial protocol bus at different data rates was implemented by an inter-integrated circuit (I^2^C) interface and serial peripheral interface (SPI). The SDRs were interfaced to the SoC/FPGA and desktop by SDR console software, such as SDR-Console V3. The experimental data were collected and processed in MATLAB, which supports scientific computing and generates the Verilog and VHDL codes for SoC/FPGA. This enables verification, data-intensive analysis and high-level design. The Analog Devices Inc. AD9363 transceiver is configured with 12-bit ADCs and DACs, supports a 325 MHz to 3.8 GHz frequency range, 20 MHz tunable channel bandwidth, low noise, high sensitivity and low errors.

**Figure 1:**
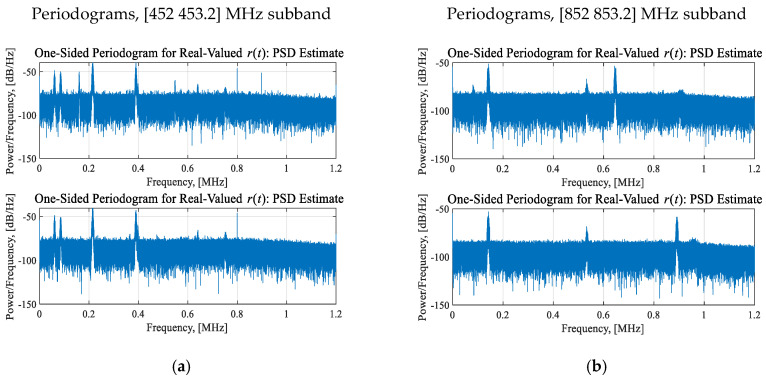
Periodograms for two subbands, [452 453.2] MHz and [852 453.2] MHz, at *t*_1_ and *t*_2_: The spectrum is evaluated for the real-valued measured signals *r*(*t*), *t_i_* ∈ [0 0.15] s, and, 360,000 samples. (**a**) Congested EMS: Periodograms for the 452 MHz subband with frequency hopping and sweeping communication. The RF signals have different modulation, characterized by the center frequency *f_ci_* ranging from 452.02 to 453.18 MHz, and the signal bandwidth *B_wi_*, *B_wi_* varying as 500 ± 50 Hz, 750 ± 250 Hz, 5 ± 1 kHz, 7.2 ± 0.5 kHz and 11 ± 1 kHz. Two RF signals with different modulation and bandwidth interfere at ~452.395 MHz; (**b**) Periodograms for the [852 853.2] MHz subband with the RF signals with corresponding center frequency *f_ci_*, and 10 ± 1 kHz bandwidth *B_wi_*.

This section documents studies for the LTE-450 and LTE-850 bands, allocated for land mobile radios, non-Federal services, cellular 3G networks, wireless LANs, Wi-Fi, Bluetooth, short-range audio and video communication, etc. The SDR outputs raw data with a data rate of 61 million samples per second, and a bandwidth of 45 MHz. We investigated multichannel 1.2 MHz subbands. The PSD was the descriptive attribute. Using the streams of the equally-divided and equally-spaced measured RF raw data for (*t*_1_, *t*_2_, …, ∆*t* = 0.15 s), *t_i_*∈[0 0.15 s], *i* = 1, 2, …, periodograms were computed. For the studied [452 453.2] MHz and [852 853.2] MHz subbands, the spectral density of RF signals with the center frequencies *f_ci_* are documented in Figure 1. In the [452 453.2] MHz subband, the RF signals exhibit different modulations. The signal bandwidth is 500 ± 50 Hz, 750 ± 250 Hz, 5 ± 1 kHz, 7.2 ± 0.5 kHz, and 11 ± 1.5 kHz. The interference and distortions are observed in the studied congested and constrained environments. For example, at 452.395 MHz, two signals are interfering.

The experimental data were analyzed using the attributes and features listed in Table 1. Consider six RF signals in the [452 452.4] MHz narrowband, as shown in Figure 1a and documented in Table 2.

**Figure 2:**
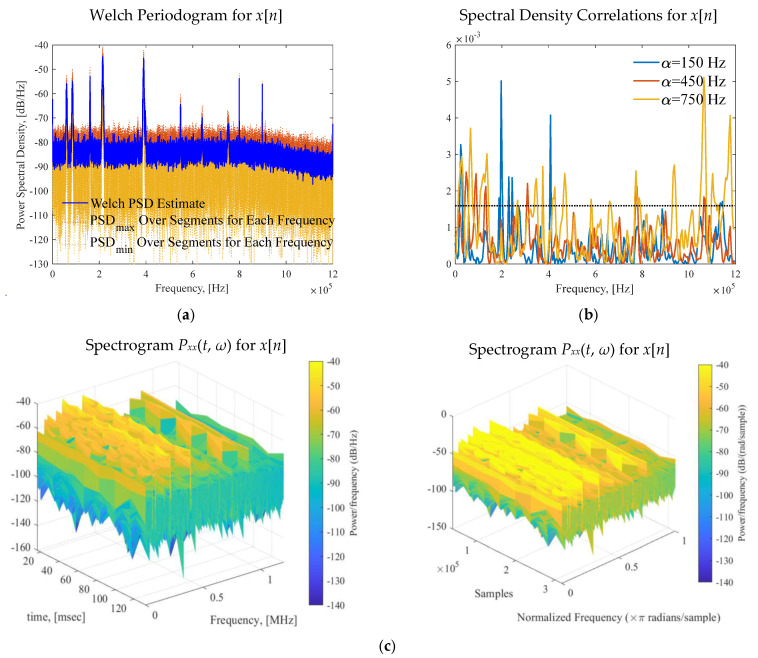
(**a**) Welch periodogram for *x*[*n*]; (**b**) Computed spectral density correlation for different cyclic frequencies; (**c**) Spectrogram: spectrum evaluation in the temporal and spectral domains.

Using the Welch method, we compute estimates for the PSD by dividing the signals into overlapping segments. The Welch periodogram reduces the variance, yielding the PSD estimates as shown in Figure 2a [40]. Fundamental, applied and experimental studies indicate nonstationary signals *x*(*t*). In addition to analysis of the marginal distribution, the processes can be analyzed by applying parametric and nonparametric quantities.

One may evaluate cyclostationarity by computing the spectral density correlation for different cyclic frequencies, assuming a stationary process, compute cyclostationarity and correlation [41]. The results are documented in Figure 2b. The PSD *S_xx_*(*f*) and autocorrelation *R_xx_*(*τ*), given by the Wiener equations, are:

(22) $$S_{xx}^{\alpha }(f)=\int_{-\infty }^{\infty }R_{xx}^{\alpha }(\tau )e^{-j2\pi f\tau }d\tau ,\;R_{xx}^{\alpha }(\tau )=\int_{-\infty }^{\infty }S_{xx}^{\alpha }(f)e^{j2\pi f\tau }df,$$

where *α* is the cycle frequency at which autocorrelation is computed.

Using the expectation operator E, the autocorrelation of *x*[*n*] is:

(23) $$R_{xx}(\tau )=E[x(t)x^{*}(t-\tau )],\;R_{xx}(\tau )=\frac{1}{N}\sum_{n=-N/2}^{N/2}x[n+\frac{1}{2}\tau ]x^{*}[n-\frac{1}{2}\tau ].$$

One obtains the following expression:

(24) $$R_{xx}^{\alpha }(\tau )=\frac{1}{N}\sum_{n=-N/2}^{N/2}x[n+\frac{1}{2}\tau ]x^{*}[n-\frac{1}{2}\tau ]e^{-j2\pi \alpha n}.$$

Figure 2b documents the cyclic spectral periodicity and coherence for different values of *α*. The experimental results imply that the channel-carrier RF signals and interference are nonstationary processes.

The spectrogram *P_xx_*(*t*, *ω*) quantitatively defines the time-varying energy content of *x*(*t*). The exhibited time-varying spectral characteristics are evaluated. The spectrogram for the [452 453.2] MHz subband is documented in Figure 2c. One finds the time-varying SNR, which affects the data rate, bandwidth, bit error rate, latency, etc. Consistent analysis in temporal and spectral domains yields high-fidelity evaluation of the EMS, supports detection of interference, as well as detects adaptive spot and barrage jamming.

**Figure 3:**
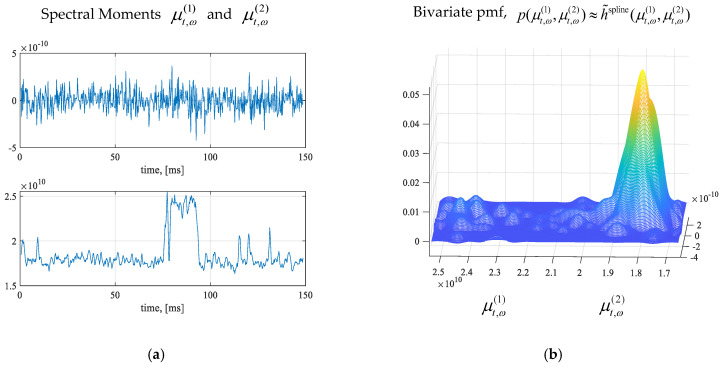
(**a**) Spectral moments $\mu_{t,\omega }^{\left(1\right)}$ and $\mu_{t,\omega }^{\left(2\right)}$; (**b**) Bivariate pdf $p(\mu_{t,\omega }^{\left(1\right)},\mu_{t,\omega }^{\left(2\right)})$.

In a narrowband, one may map nonstationary processes as stationary by using the method of differencing. The difference–stationary stochastic processes are used, achieving an acceptable degree of stationarity. Characterize the RF signals in the multi-band spectrum using the naïve Bayes models and Jaccard index. In the studied narrowband, using the threshold on the signal power, six high-power signals were used, which are documented in Figure 1a and Table 2. We studied the observed bandwidth-clustered RF signals with varying power, gradients, frequency, covariance and correlation. That is, identify and apply descriptive and informative features and indicators to explicitly characterize signals within the spectrum. We used the signal bandwidth *B_w_*, PSD *S_xx_*(*ω*), gradient of power variations $\nabla_{\omega }S_{xx}(\omega )$, and probability characteristics. With a focus on high-fidelity analysis, one considers the transceiver modalities, temporal modes, etc. The signal corner frequency *f_c_* may not be considered as an attribute due to frequency hopping and sweeping. To characterize instantaneous variations in spectra, the spectral moments are used. Using (5), the computed spectral moments $\left(\mu_{t,\omega }^{\left(1\right)},\mu_{t,\omega }^{\left(2\right)}\right)$ are documented in Figure 3a. The bivariate pmf for $p(\mu_{t,\omega }^{\left(1\right)},\mu_{t,\omega }^{\left(2\right)})$ and distributions are found by spline interpolating the normalized bivariate histograms. Figure 3b reports the computed descriptive pmf $p(\mu_{t,\omega }^{\left(1\right)},\mu_{t,\omega }^{\left(2\right)})\approx {\tilde{h}}^{\mathrm{spline}}(\mu_{t,\omega }^{\left(1\right)},\mu_{t,\omega }^{\left(2\right)})$ which yields the distribution under the applied hypotheses.

**Figure 4:**
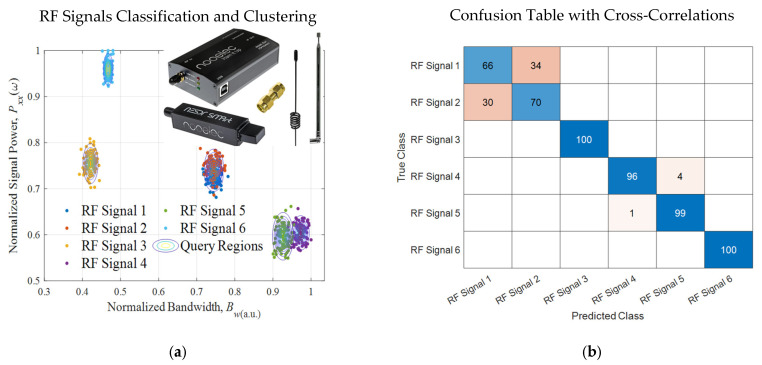
(**a**) Classification and clustering of RF signals *r*(*t*), measured by the SDR, using ${\left(B_{w},P_{xx}(\omega ),\nabla_{\omega }P_{xx}(\omega )\right)}_{a.u.}\in [0\;1]$: Signals are detected and classified with probabilities from 0.62 to 0.95 for different [0, *t*)*_l_*_=1,2,…_; (**b**) RF signals cross-correlation.

For stationary stochastic processes, the real-valued periodogram, as an attribute of the spectral density and frequency content, is the squared absolute value of the finite-time Fourier transform of a signal. The Fourier transform and periodogram are continuously differentiable functions. Furthermore, the Fourier transform and periodogram are square integrable and bounded. The periodogram for a sequence [*x*_1_, …, *x_N_*], given by (4) ${\left.S_{xx}(\omega )\right|}_{\omega =\frac{2\pi k}{N}}=\frac{1}{N}{\left.{\left|X(e^{j\omega })\right|}^{2}\right|}_{\omega =\frac{2\pi k}{N}}=\frac{1}{N}{\left|X[k]\right|}^{2}=\frac{1}{N}{\left|\sum_{n=0}^{N-1}x[n]e^{-j\frac{2\pi }{N}kn}\right|}^{2}$, is normalized. Within the spectrum, computing the area under the continuous periodogram for the detected *i*th signals with bandwidth *B_wi_*, we find the average power *P_xxi_*(*ω*, *B_wi_*) of the signal. The spectrogram yields the time-varying power of the RF signals. The amplitude-shift keying, phase-shift keying (PSK), quadrature PSK, differential PSK, frequency-shift keying, quadrature amplitude modulation, orthogonal frequency-division multiplexing modulation and other modulation schemes yield distinguishable (*B_w_*, *P_xx_*) and gradient of power $\nabla_{\omega }P_{xx}(\omega )$. To ensure consistency, the arbitrary units (a.u.) are used. For the signals in the subband, consider ${\left(B_{w},P_{xx}(\omega ),\nabla_{\omega }P_{xx}(\omega )\right)}_{a.u.}\in [0\;1]$. Figure 4a documents $\left(B_{w},P_{xx}(\omega )\right)$. The nearest neighbors and naïve Bayesian models are applied.

The results are visualized as follows:

1. Query ellipse $\frac{x_{i}}{a_{i}^{2}}+\frac{y_{i}}{b_{i}^{2}}=1$ for two features;
2. Three-dimensional closed ellipsoid region $\frac{x_{i}}{a_{i}^{2}}+\frac{y_{i}}{b_{i}^{2}}+\frac{z_{i}}{c_{i}^{2}}=1$,(*a_i_*,*b_i_*,*c_i_*)>0 for the following three features ${\left(B_{w},P_{xx}(\omega ),\nabla_{f}P_{xx}(\omega )\right)}_{a.u.}$;
3. Ellipsoids in an *n*-dimension. A quadric hypersurface is $\sum_{l=1}^{n}\frac{{\left(x_{i,l}-p_{i,l}\right)}^{2}}{a_{i,l}^{2}}=1$, where *p_i_*_,*l*_ are the center coordinates, and, *a_i_*_,*l*_ are the length of the semi-axis.

**Figure 5:**
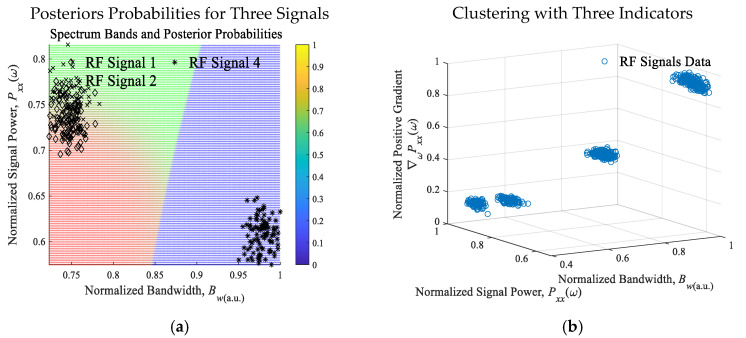
(**a**) Posteriors probabilities for three RF signals; (**b**) Classification and clustering using ${\left(B_{w},P_{xx}(\omega ),\nabla_{\omega }P_{xx}(\omega )\right)}_{a.u.}$.

Using the reported algorithm, six signals were detected, identified and characterized. Adequate classification probability was achieved. Figure 4a depicts the query regions. For different measurements at (*t*_1_, *t*_2_, …, ∆*t*), the classification accuracy for six signals varies between 62% and 95% due time-varying ${\left(B_{w},P_{xx}(\omega ),\nabla_{\omega }P_{xx}(\omega )\right)}_{a.u.}$. The RF signals 1 and 2 exhibit similar attributes and features and are likely identical to the two-channel redundant communication by the same transmitter. Cross-validation of the models was performed. The signals were then tabulated and classified, as reported in Figure 4 and Figure 5. Figure 4a illustrates the RTL-SDR dongle, connected to the laptop/desktop or SoC/FPGA using a USB port and WinUSB/Zadig driver. The frequency range is from 500 kHz to 1750 MHz. Using the L1-norm to define the loss function $J={\left\|x_{i}\right\|}_{1}+{\left\|y_{i}\right\|}_{1}$, the maximum cross-validation loss is 0.205. We achieved an adequate accuracy in detection, characterization, classification and clustering. The confusion table is documented in Figure 4b. The *Büchi automata* scheme yields consistent accuracy with latency less than 0.053 s. The computed posteriors probabilities, shown in Figure 5a, yield an accuracy in classification from 62% to 95% for signals 1 and 2, and, 94 to 100% for other RF signals, as documented in Figure 5b. For the considered case studies in the studied commercial RF bands, the results [22,23] yield high misclassification, except for signals 1 and 2. The feedforward fully connected neural network was evaluated. Learning algorithms, models and scalable machine reasoning schemes were studied and substantiated.

## 5. Discussion

Spectrum and signals are not stationary, and data heterogeneity significantly affects pattern recognition, EMS evaluation, as well as signal characterization and classification. Particular challenges arise in communication and information sharing networks due to interference, perturbations and jamming. By means of a proposed analysis and machine reasoning frameworks, this paper fosters new methodologies to solve some open problems. The proposed quantitative attributes and finite-dimensional aggregated features in temporal, spectral and probability domains ensure algorithms consistency, convergence and robustness. The multidomain analysis advances knowledge and fosters a foundation for future research. The results are verified in the congested multiband spectra. The RF signals and interference can be detected and classified. Analysis and evaluation of EMS in temporal and spectral domains support detection of adaptive spot and barrage jamming. The technology roadmap is focused on computationally effective and scalable algorithms and machine reasoning with real-time processing capabilities to support resilient communication. Experimental studies and benchmarking yield formative analysis, summative assessment and comparative evaluation of available methodologies. The performance metrics and transformative solutions demonstrate advantages and effectiveness of the proposed algorithms.

## 6. Conclusions

Congested, contested and constrained electromagnetic spectra were investigated. Descriptive characteristics were devised and used, stipulating the finite-dimensional domain-specific attribute and feature spaces. We investigated data-driven formative analysis and machine reasoning algorithms to support resilient and reconfigurable communication. Our motivation was to research inroads in characterization of the EMS, classification of RF signals, as well as detection of perturbations and jamming. These studies may support mitigation of interference, enable adaptation to dynamic EMS, optimize spectrum sharing, as well as control reconfigurable RF channels division and scheduling. Our findings may foster scalable solutions for legacy and new generations of SDRs and software defined mobile networks, supporting modernization and advancements of future communication systems. The aforementioned developments contribute to robust and seamless information sharing. The reported findings are crucial to advance knowledge, verify hypotheses, and evaluate dynamic EMS.

## Figures and Tables

**Table 1:** Attributes and features.

Attributes	Features: Domain-Specific Quantitative Characteristics, Transforms and Operators on Attributes and Metrics
1. RF signals modulation and polarization.	1. Computed spectrum characteristics, periodograms, spectrograms, PSD, gradient estimates, etc.
2. Bands, wavelength, center frequency *f_c_* and bandwidth *B_w_.*	2. Temporal and probabilistic characteristics on (*f_c_*,*B_w_*), including statistical regularity.
3. Signal (carrier, noise, interference and jamming) strength, power spectral density, signal-to-noise ratio, etc.	3. Temporal, spectral and probabilistic characteristics, such as: Finite-dimensional transforms, distributions, pmf, cdf, variance, correlation, spectral moments, etc.

**Table 2:** Six RF Signals in the [452 452.4] MHz subband, characterized by the center Frequency, PSD and bandwidth (*f_c_*, *S_xx_*, *B_w_*).

RF Signals, (*f_c_*, *S_xx_*, *B_w_*)	
1	*f_c_* = 452.0602 MHz, *S_xx_* = −48.58 dB/Hz, *B_w_* = 9.4 kHz.
2	*f_c_* = 452.0846 MHz, *S_xx_* = −49.75 dB/Hz, *B_w_* = 9.4 kHz.
3	*f_c_* = 452.1604 MHz, *S_xx_* = −50.23 dB/Hz, *B_w_* = 5.3 kHz.
4	*f_c_* = 452.2154 MHz, *S_xx_* = −39.74 dB/Hz, *B_w_* = 12.3 kHz.
5	*f_c_* = 452.3907 MHz, *S_xx_* = −39.38 dB/Hz, *B_w_* = 11.7 kHz.
6	*f_c_* = 452.3983 MHz, *S_xx_* = −63.84 dB/Hz, *B_w_* = 5.9 kHz.

## Data Availability

The data and results are reported in the paper.
